# Exploring Germplasm Diversity to Understand the Domestication Process in *Cicer* spp. Using SNP and DArT Markers

**DOI:** 10.1371/journal.pone.0102016

**Published:** 2014-07-10

**Authors:** Manish Roorkiwal, Eric J. von Wettberg, Hari D. Upadhyaya, Emily Warschefsky, Abhishek Rathore, Rajeev K. Varshney

**Affiliations:** 1 International Crops Research Institute for the Semi-Arid Tropics (ICRISAT), Hyderabad, Andhra Pradesh, India; 2 Department of Biological Sciences, Florida International University, Miami, Florida, United States of America; 3 Center for Tropical Plant Conservation, Fairchild Tropical Botanic Garden, Miami, Florida, United States of America; Nanjing Agricultural University, China

## Abstract

To estimate genetic diversity within and between 10 interfertile *Cicer* species (94 genotypes) from the primary, secondary and tertiary gene pool, we analysed 5,257 DArT markers and 651 KASPar SNP markers. Based on successful allele calling in the tertiary gene pool, 2,763 DArT and 624 SNP markers that are polymorphic between genotypes from the gene pools were analyzed further. STRUCTURE analyses were consistent with 3 cultivated populations, representing kabuli, desi and pea-shaped seed types, with substantial admixture among these groups, while two wild populations were observed using DArT markers. AMOVA was used to partition variance among hierarchical sets of landraces and wild species at both the geographical and species level, with 61% of the variation found between species, and 39% within species. Molecular variance among the wild species was high (39%) compared to the variation present in cultivated material (10%). Observed heterozygosity was higher in wild species than the cultivated species for each linkage group. Our results support the Fertile Crescent both as the center of domestication and diversification of chickpea. The collection used in the present study covers all the three regions of historical chickpea cultivation, with the highest diversity in the Fertile Crescent region. Shared alleles between different gene pools suggest the possibility of gene flow among these species or incomplete lineage sorting and could indicate complicated patterns of divergence and fusion of wild chickpea taxa in the past.

## Introduction

Many crops that are grown across multiple regions have limited genetic diversity due to bottlenecks from domestication, selective breeding and in some taxa, natural processes [1]–[4]. Recurrent selection of improved cultivars over multiple generations results in an increasingly narrow genetic base for a crop, making it more vulnerable to disease and limiting its adaptability. Such genetically depauperate crops could have disastrous consequences in the face of emerging diseases and climate change [5], [6]. Recent applications of genome mapping suggest that the genetic diversity stored in germplasm banks can be utilized with a much higher level of efficiency than previously imagined [6], [7]. This is particularly true for self-pollinated crops like chickpea (*Cicer arietinum*). During the past few decades, our understanding of the importance of plant genetic resources and the need to conserve them has grown [8], and wild relatives are now commonly seen as a key source of genetic diversity that can be used to increase diversity in breeding material [7], [9]. Diversity estimates of germplasm collections have not been universally performed to assess the scope of diversity available in existing collections. Such estimates are critical for providing insight into efforts to introgress wild germplasm into elite lines, and for guiding future collections of wild germplasm [10].

In order to make more efficient use of wild relatives, we need improved classifications of their relationship to crop material and to other wild species [11]. Characterizing patterns of diversity within the secondary and tertiary gene pools [12] can provide insight into which subdivisions of germplasm collections contain wild material that is most likely to increase diversity and can guide the use of wild material in breeding efforts. Although wild material is rarely used in breeding programs due to agronomically poor traits, it remains a chief reservoir for many disease and abiotic stress resistance traits. Effective characterization of wild material can facilitate its more effective use [13].

Chickpea is an important crop in semi-arid tropical regions such as South Asia and Eastern & Southern Africa, Mediterranean regions, and cool temperate areas [14]. Globally, chickpea is the second most widely consumed legume after beans (*Phaseolus*) [15]. Lack of genetic diversity has long been a critical problem for chickpea breeding [16], limiting efforts to improve resistance to diseases like *Ascochyta* blight and *Fusarium* wilt, pod borer insects, and tolerance to abiotic stresses like terminal drought, high and low temperatures [17], [18]. Chickpea reference set has also been used to understand the available diversity for stress responsive genes [19]. Widening the genetic diversity of cultivated chickpea is dependent on the introduction of alleles controlling the traits of interest from wild germplasm [1]. Currently chickpea’s immediate ancestor, *C. reticulatum*, and its interfertile sister species *C. echinospermum,* is the main source of new variation, although introgression is possible from the more distantly related gene pools with greater effort [20].

Cultivated chickpea first appears in the archaeological record some 6.6–7.2 thousand years ago in Syria [21], [22]. The immediate wild relatives (*C. reticulatum* and *C. echinospermum*) of chickpea are restricted to southeastern Turkey [1]. Domestication is thought to have happened earlier, as much as 10.5 thousand years ago, concurrent with or soon after the domestication of other Fertile Crescent crops such as wheat, barley, pea, and lentil. Domesticated chickpea was likely brought to Syria about 7,000 years ago, while records for the dates of introduction into East Africa and the Indian subcontinent are limited [22]. Abbo and co-workers [1], [23] have speculated that chickpea is particularly genetically depauperate because it may have gone through four distinct bottlenecks: modern breeding, domestication, a shift early in its cultivation from a winter annual phenology to a spring phenology, and wild relatives (particularly *C. reticulatum* and *C. echinospermum*) that have a narrow geographic distribution compared to other crops domesticated in the Fertile Crescent. The shift in phenology may have accompanied the introduction of other crops such as sesame and sorghum that are summer annuals [24]. Breeding for preferred phenotypes, such as seed colour and shape, may exacerbate chickpea’s narrow genetic base and may be one of the key reasons for slow progress in yield improvement and increased tolerance to various biotic and abiotic stresses. Based on seed shape, size and colour, chickpea is classified into two seed types, kabuli and desi. The kabuli chickpea is characterized by a larger, cream-coloured seed with a thin seed coat, while the desi seed type has a smaller, darker coloured seed with a thick seed coat. In addition, a third seed type, designated as intermediate or pea-shaped, is characterized by medium to small size and round, pea-shaped seeds [25].

Single nucleotide polymorphism (SNP) markers have become the markers of choice for various genome wide analyses because they are widespread across genomes, accurate and reproducible, and well suited to automated detection [26]. A range of low- to high-throughput SNP genotyping platforms have become available to make SNP genotyping cost-effective such as BeadXpress, KBioscience Competitive Allele-Specific Polymerase chain reaction (KASPar) assays, and GoldenGate assays from Illumina Inc. [27], [28]. In addition, another high-throughput marker system, Diversity arrays technology (DArT), has proven useful for screening large numbers of loci in crops with low genetic diversity, and DArT markers for chickpea have recently been developed [29].

The present study is focused on the assessment of relationships in a diversity panel of chickpea which includes breeding material from the three seed types (kabuli, desi, and pea-shaped) and wild species from the primary, secondary, and tertiary gene pools using KASPar technology and hybridization based DArT arrays for high-throughput SNP genotyping. We examined the level of genetic differentiation among these groups of genotypes and assessed how segregating variation is spread across the genome of chickpea.

## Materials and Methods

### Germplasm and DNA isolation

A diverse set of 94 chickpea genotypes (Table S1) including 66 cultivars and landraces (23 desi, 41 kabuli, and 2 pea-shaped seed type genotypes) and 28 genotypes from 9 wild species including genotypes from primary, secondary and tertiary gene pool was selected as a diversity panel for assessment from the ICRISAT germplasm collection [30].

Total genomic DNA was isolated from 10–12 leaves of two week old plants following a modified CTAB protocol as described in Cuc et al. [31]. Only one plant per accession was used for DNA isolation. DNA quality and quantity for each sample was assessed on 0.8% agarose gel.

### Genotyping

SNPs were identified using four different approaches: Solexa/Illumina sequencing, mining of Sanger Expressed Sequence Tags (ESTs), allele-specific sequencing of candidate genes, and allele-specific sequencing of tentative orthologous genes (TOGs) as described by Hiremath et al. [28]. In total, 2,486 SNPs were used for validation and development of KASPar assays by KBioscience, of which 2,005 (80.6%) assays could be validated and designated as Chickpea KASPar Assay Markers (CKAMs) [28]. A subset of highly polymorphic 651 CKAMs was used for genotyping using KASPar assays. In addition, this diverse set was also genotyped with high-density DArT array with 15,360 DArT clones as described in Thudi et al. [29].

### Data Analysis

**Figure 1:**
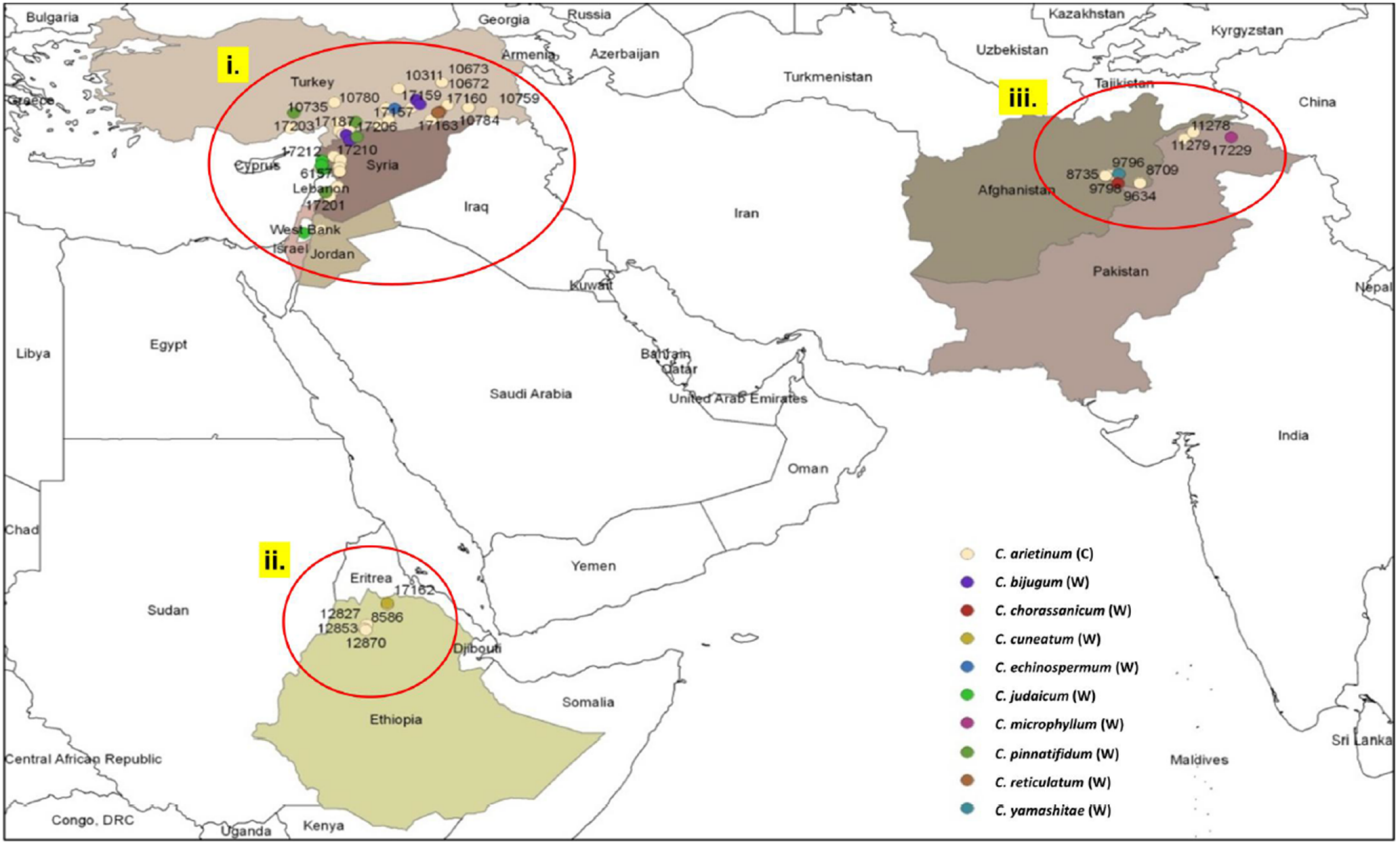
Geographic locations of cultivated and wild *Cicer* species collection sites (C: Cultivated; W: Wild) i. Fertile Crescent; ii. Ethiopia; iii. Central Asia.

The germplasm was divided into three different clusters based on geographical origin, namely the Fertile Crescent, Central and South Asia, and Ethiopian Highlands (Figure 1). Additionally, germplasm was classified based on gene pools (primary, secondary, and tertiary) [32], seed type (desi, kabuli, and pea-shaped) and wild vs. cultivated species. The purpose of these different divisions of the data was to determine the scale over which genetic variation is present in the germplasm collection. In order to assess hierarchical levels of variation within and between different sub-groups, DArT and SNP genotyping data were analyzed separately. AMOVA was conducted on the DArT markers based on the hierarchical model and permutational procedures of Excoffier et al. [33] to assess the level of variation among these wild and domesticated groups. We implemented AMOVA in GenAlEx 6.5 [34], [35] and Arlequin [36]. AMOVA analysis with populations nested within regions was also performed to examine the distribution of variation and differential connectivity among populations (PhiPT; an analogue of Fst, i.e., genetic diversity among populations). In addition, Shannon information index (measure of species diversity in a population) was calculated for all the population using GenAlEx 6.5. This index provides important information about rarity and commonness of species in a community by taking relative abundances of different species into account [34], [37].

A separate AMOVA was performed on the SNP data to assess variation within and among desi, kabuli, and pea-shaped seed types. In both AMOVAs, we assessed genetic variation within groups (Fct), within populations (Fst), between populations within a group (Fsc), population polymorphism, and Nei’s genetic distance and gene flow (Nm) using GenAlEx v.6.41 [34], [35] and Arlequin [36]. For each group presence of private alleles (np), percentage of polymorphic loci (%p), the average number of alleles per locus (k), the expected heterozygosity (He), and unbiased expected heterozygosity (UHe) across different subgroups (i.e., wild species *vs* cultivated with the DArT markers and seed type with the SNP markers) was calculated. The polymorphism information content (PIC) values for SNP and DArT markers across 94 diverse genotypes were calculated by using PowerMarker software [38].

STRUCTURE 2.3 [39] was used to estimate the number of natural genetic groups (K), the distribution of individuals among these groups, and to assign individual genotypes to a specified number of groups “K” based on membership coefficients calculated from the genotype data. This approach is an important complement to the hierarchical division of the germplasm (see above), as it can determine the number of groups best supported by the DArT and SNP data. DArT data was converted in to psuedo-diploid format by assigning a row of missing data to each individual so that it could be analysed with STRUCTURE. We assessed a range of population numbers from K = 1 to K = 15 using a burn-in period of 50,000 steps followed by 500,000 MCMC (Monte Carlo Markov Chain) replicates with 3X iterations, assuming admixture and correlated allele frequencies. Due to missing SNP calls in the wild material, data from wild material was separated from that of cultivated material and a separate STRUCTURE analysis of cultivated material alone was performed using SNP markers. In order to compliment the STRUCTURE analyses, pair-wise genetic differentiation between individuals was calculated from the DarT markers, which was used in principal coordinate analysis (PCoA), implemented in GenAlEx 6.5. These analyses labelled the material based on its source region: the Fertile Crescent, Central Asia, and the Ethiopian highlands.

A complementary approach to assessing relationships among taxa is a phylogenetic analysis. Distance-based phylogenetic analysis of SNP data was performed using the software package Geneious v. 7.0.6 (Biomatters) (http://www.geneious.com). A cladogram was produced using unweighted pair-group method with arithmetic mean (UPGMA) cluster analysis under the Jukes-Cantor genetic distance model with 100 bootstrap replications. The consensus tree was then rooted with the clade of individuals from the tertiary gene pool.

## Results

### Marker attributes

**Figure 2:**
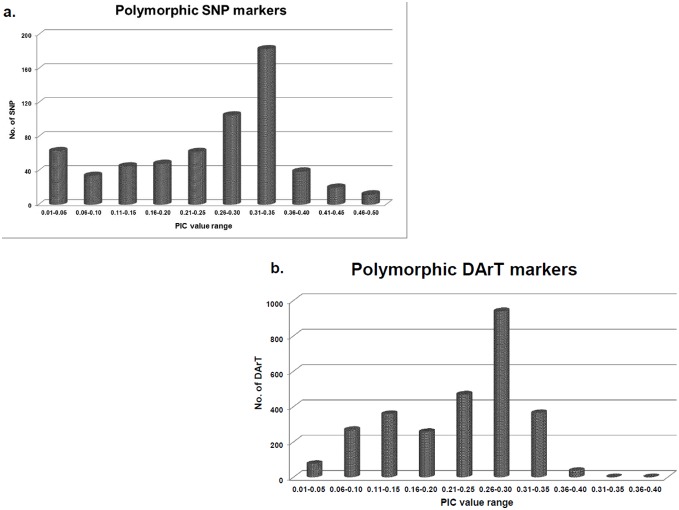
Polymorphism information content (PIC) value of markers used in study. a. PIC value of SNP markers used for diversity analysis. b. PIC value of DArT markers used for diversity analysis.

In total, 651 SNP markers using KASPar assays and DArT arrays were used for genotyping the set of 94 diverse chickpea genotypes. This set includes 66 cultivated chickpea genotypes and 27 wild relatives representing eight wild *Cicer* species from primary, secondary, and tertiary gene pools along with one perennial wild chickpea genotype. The genotypes were carefully selected to represent geographical areas with the most phenotypic diversity: the Fertile Crescent, Central Asia, and the Ethiopian highlands (Figure 1). SNP markers were highly polymorphic across this diverse set and a total of 611 SNPs were found polymorphic. The polymorphic information content (PIC) value ranged from 0.02 to 0.50 across these 94 genotypes with mean PIC value of 0.23 (Figure 2a). Although these SNPs were highly polymorphic, in many cases SNPs could not be called for wild chickpea genotypes (Table S2). SNPs were developed using cultivated chickpea and later used for genotyping the wild species, which may account for the greater number of missing loci in the tertiary gene pool and the bimodal distribution of PIC values.

To overcome the issue of missing data in the wild material and to compliment the SNP data, the set was genotyped using high density DArT arrays with 15,360 clones [29]. A total, 5,257 DArT markers were polymorphic across 94 lines. Of these, a subset of 2,763 markers was selected for use in the present study based on the presence of the allele in wild chickpea (tertiary gene pool). PIC for these 2,763 DArT markers ranged from 0.02 to 0.37, with an average of 0.22 across the 94 genotypes (Figure 2b) (Table S3).

### Differences among the wild species and cultivated germplasm

**Figure 3:**
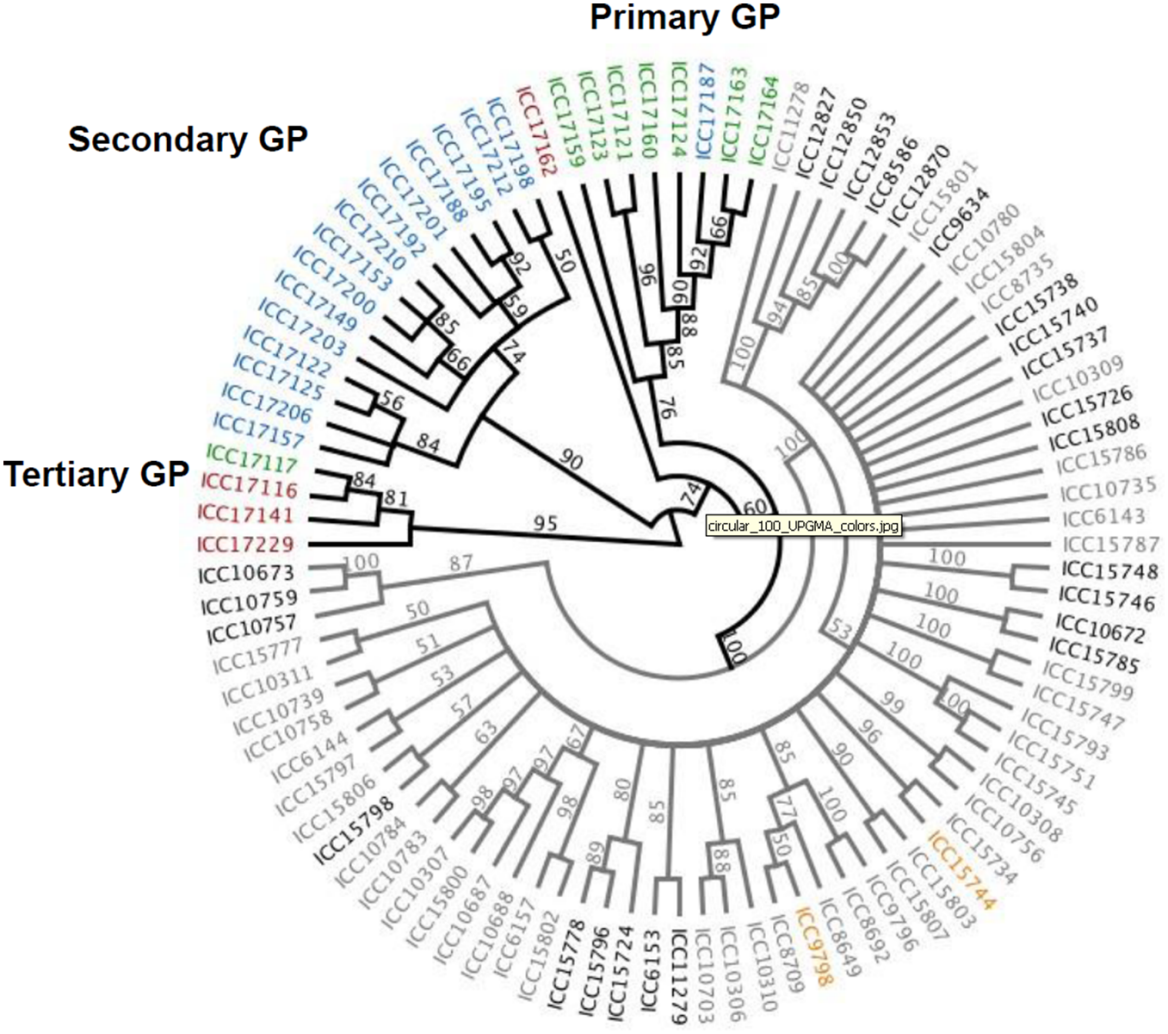
UPGMA tree of pairwise relatedness of cultivated (grey branches) and wild (black branches) chickpea. Genepools and seed types are represented by the following colors: primary, green; secondary, blue; tertiary, red; pea-shaped, orange; kabuli, grey; and desi, black.

The chickpea diversity panel used in the present study is comprised of 94 genotypes from 9 wild species (8 annual and 1 perennial) and cultivated species (*C. arietinum*). DArT data was used to understand the diversity and genetic architecture of the germplasm. As expected, wild species genotypes had higher levels of polymorphic markers (99.60%) compared to cultivated genotypes (35.79%) (Table 1). A UPGMA tree was constructed based on pairwise genetic distances using the SNP markers to understand the relationships between the genotypes from wild and cultivated species (Figure 3). Two major groups were identified by this analysis, separating wild from cultivated genotypes. Cultivated and wild species genotypes from the primary gene pool were grouped in one cluster (Figure 3). However, genotypes from the chickpea ancestor, *C. reticulatum,* were interspersed with those from cultivated individuals, consistent with a close relationship between ancestral and cultivated chickpea. Genotypes from the secondary gene pool species were found to cluster together, as were genotypes from the tertiary gene pool.

**Table 1:** Assessment of genetic diversity across groups of wild and cultivated chickpea using DArT markers.

	Polymorphic marker (%)	N	Na	Ne	I	He	UHe
**Cultivated**	35.79	63.40**1**±0.04	1.219±0.013	1.096±0.004	0.113±0.004	0.068±0.002	0.068±0.002
**Wild**	99.60	27.143±0.023	1.996±0.001	1.766±0.005	0.607±0.002	0.421±0.002	0.429±0.002
**Mean**	67.70	45.272±0.245	1.607±0.008	1.431±0.005	0.360±0.004	0.244±0.003	0.249±0.003

No. of polymorphic alleles (N), No. of Different Alleles (Na), No. of Effective Alleles (Ne, = 1/(Sum pi∧2)), Shannon’s Information Index (I = −1 * Sum (pi * Ln (pi))), Expected Heterozygosity (He = 1−Sum pi∧2) and Unbiased Expected Heterozygosity (UHe = (2N/(2N−1)) * He).

**Figure 4:**
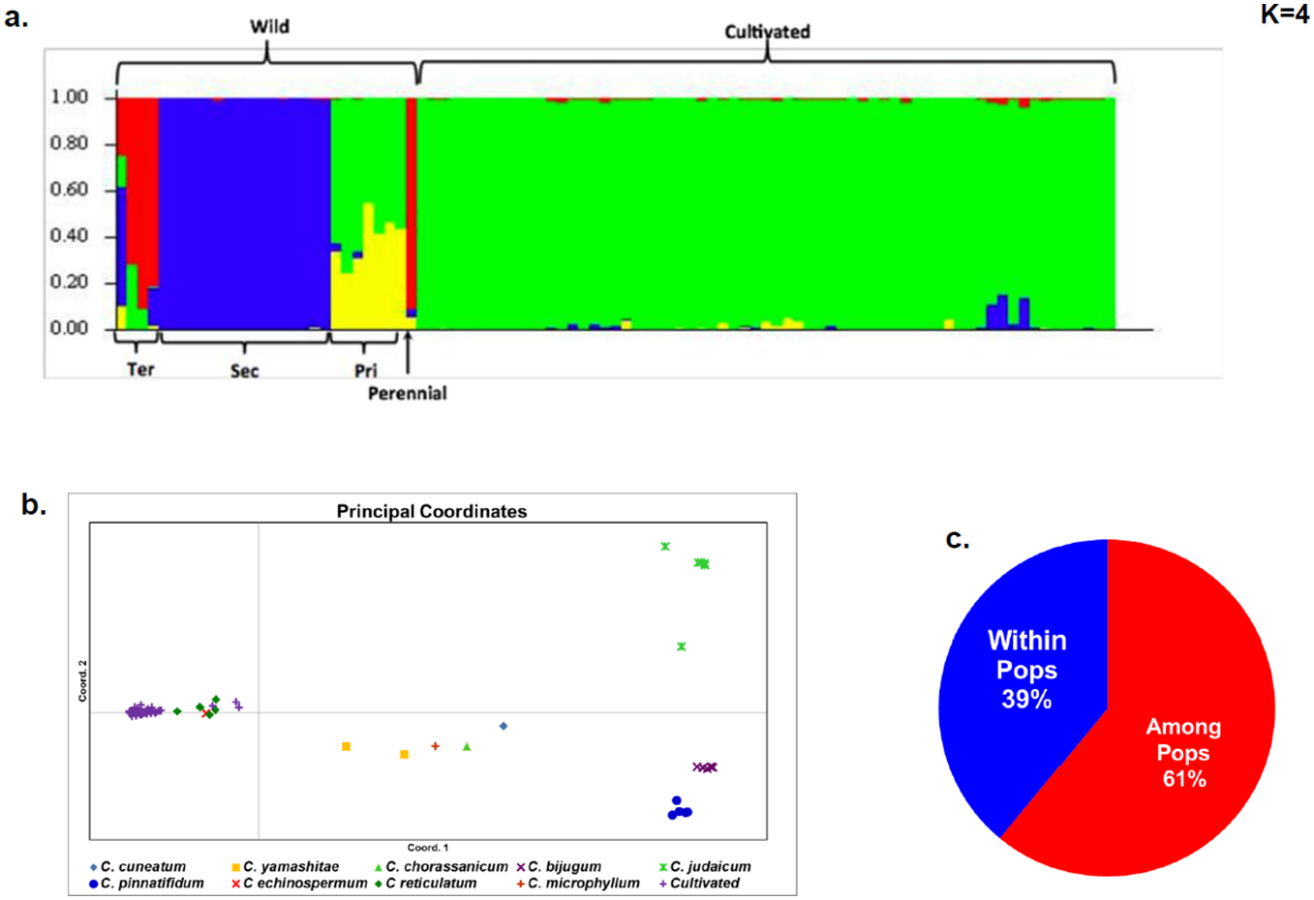
Population structure analysis using STRUCTURE of *Cicer* accessions. a. Structure showing distinct group of wild and cultivated species; wild further classified in primary (Pri), secondary (Sec) and tertiary (Ter) gene pool species. b. Principal coordinates analysis among wild and cultivated species. c. Analysis of molecular variance between and among wild and cultivated species genotypes.

In parallel, STRUCTURE was also used to understand the clustering between cultivated and wild species genotypes. With the DArT data, STRUCTURE resolved four clusters using the Evanno method (Figure 4a). This grouping indicates a substantial difference between wild and cultivated material, as well as major differences within the wild material. These results suggest that there are three major groups among the wild material (Figure 4a), corresponding to different gene pools. Individuals in the tertiary gene pool are represented largely as one cluster with admixture; although these individuals represent several species (with the capacity to hybridize) and are certainly not a homogenous group, they do cluster together. The perennial species in the tertiary gene pool, *C. microphyllum,* appears admixed with the primary gene pool. However, this could be due to its closer phylogenetic relationship to *C. reticulatum* or accidental gene flow in the germplasm collection. The secondary gene pool, with the closely related and interfertile species of *C. pinnatifidum*, *C. bijugum* and *C. judaicum* formed one tight cluster. The immediate ancestors of the crop, *C. reticulatum* and *C. echinospermum,* show up as a group with substantial admixture with the cultivated individuals. This could represent the derivation of the crop, and could also represent introgression from the crop to the wild species (or artefacts of maintenance in germplasm facilities). The cultivated accessions of *C. arietinum* showed little admixture with the wild material in this analysis.

In addition, principal coordinate analysis, which was performed as a complementary approach to display clustering of genotypes, separated cultivated genotypes from wild species genotypes. Few genotypes of the wild chickpea clustered with cultivated material. Those wild genotypes that did cluster were *C. reticulatum,* the likely progenitor of cultivated chickpea (Figure 4b). The PCoA showed substantial differences among the wild material; *C. reticulatum* and *C. echinospermum* genotypes clustered with closely related cultivated material (Figure 4b). However, the closely related species from the secondary gene pool clustered individually rather than all clustering together. Furthermore, genotypes from a species in the tertiary gene pool, *C. yamashatae*, clustered more closely with the primary gene pool than did the species of the secondary gene pool. AMOVA partitioned 39% of variation between wild and cultivated groups and 61% of variation segregating within groups (Figure 4c).

### Genetic diversity among the genotypes from wild chickpea

The present study included analysis of 28 chickpea genotypes from nine wild species including genotypes from primary, secondary, tertiary gene pools and one individual of a perennial species, *C. microphyllum*. Genotyping using SNP markers resulted in high rates of failed SNP allele calls and null alleles. We therefore used DArT data to estimate the genetic diversity and relationships among the cultivated and wild species genotypes for primary, secondary and tertiary gene pools. AMOVA of wild species genotypes indicated that 31% of variation was found among the species while 69% of variation was observed within the species. Genetic distance between populations (primary, secondary and tertiary) was calculated based on Nei’s genetic distance. As expected, higher similarity was observed between the primary and secondary gene pools (Nei’s genetic distance 0.15), while greater distance was observed between primary and tertiary gene pools (Nei’s genetic distance 0.69). Furthermore, a greater distance was observed between the secondary and tertiary gene pools than between the primary and secondary gene pools, which suggests that genotypes from the primary and secondary gene pools are more closely related to each other than to the tertiary gene pool. Across all wild material, numbers of effective alleles and values of heterozygosity were much higher than in the crop material. Within the wild material, the secondary gene pool had the greatest diversity, with highest effective allele estimates and highest heterozygosity (Table 2).

**Table 2:** Assessment of genetic diversity across wild germplasm using DArT markers.

	Polymorphic marker (%)	N	Na	Ne	I	He	UHe
**Primary GP**	74.34	6.915±0.006	1.743±0.008	1.521±0.007	0.429±0.005	0.294±0.004	0.317±0.004
**Secondary GP**	95.04	15.519±0.014	1.935±0.006	1.63±0.007	0.521±0.004	0.353±0.003	0.365±0.003
**Tertiary GP**	10.82	3.754±0.011	0.86±0.011	1.072±0.004	0.061±0.003	0.041±0.002	0.048±0.003
**Mean**	60.07	8.729±0.055	1.513±0.007	1.407±0.004	0.337±0.003	0.229±0.002	0.243±0.002

No. of polymorphic alleles (N), No. of Different Alleles (Na), No. of Effective Alleles (Ne, = 1/(Sum pi∧2)), Shannon's Information Index (I = −1* Sum (pi * Ln (pi))), Expected Heterozygosity (He = 1−Sum pi∧2) and Unbiased Expected Heterozygosity (UHe = (2N/(2N−1)) * He).

In the PCoA of the wild material alone (Figure S1a), a few genotypes from the primary gene pool clustered with the tertiary gene pool genotypes. Other genotypes from the primary gene pool clustered with the secondary gene pool. In parallel, we performed a STRUCTURE analysis on the 28 wild species genotypes using DArT markers. The STRUCTURE results complemented the observation from PCoA and diversity analysis (Figure S1b). We selected K = 2 based on Evanno method. The first cluster corresponds to the primary gene pool, while the second cluster corresponds to the secondary gene pool. The tertiary gene pool was admixed, likely representing the great diversity in those disparate species.

### Genetic diversity among phenotypic classes of cultivated chickpea

Diversity among the 66 cultivated genotypes was assessed using both the DArT and SNP markers. These 66 genotypes were classified in three sub-groups based on seed type, *i.e.* desi, kabuli and pea-shaped. SNP markers were used in the program STRUCTURE to resolve differences among phenotypic classes of cultivated chickpea. Three groups of the cultivated material (K = 3) were observed, with most individuals demonstrating substantial admixture (Figure S2a). Genetic diversity among the phenotypic classes was also assessed using DArT and SNP markers (Table 3). The number of effective alleles (Ne) and heterozygosity (He) were very similar among the phenotypic classes (with overlapping standard deviations around their means), and all values were low (i.e., <1.1 for Ne, and <0.1 for He). Hierarchical AMOVA using both SNP and DArT data provided similar results. More than 90% of variation was observed within these phenotypic classes, while only about 10% variation was reported among these different populations (Figure S2b).

**Table 3:** Assessment of genetic diversity across chickpea germplasm based on seed type.

Marker Type	Seed type	Polymorphic marker (%)	N	Na	Ne	I	He	UHe
**SNP**	**kabuli**	15.22	41.747±0.026	1.152±0.014	1.046±0.006	0.052±0.006	0.031±0.004	0.032±0.004
	**desi**	4.81	23.897±0.014	1.048±0.009	1.031±0.006	0.025±0.005	0.017±0.003	0.018±0.003
	**pea**	2.56	1.901±0.012	1.026±0.006	1.026±0.006	0.018±0.004	0.013±0.003	0.017±0.004
	**Total**	7.53	22.515±0.377	1.075±0.006	1.034±0.004	0.032±0.003	0.02±0.002	0.022±0.002
**DArT**	**kabuli**	18.64	39.101±0.028	0.987±0.012	1.06±0.003	0.065±0.003	0.04±0.002	0.041±0.002
	**desi**	26.89	22.348±0.019	1.094±0.013	0.11±0.004	0.109±0.004	0.069±0.003	0.071±0.003
	**pea**	0.54	1.952±0.004	0.725±0.009	1.004±0.001	0.003±0.001	0.002±0.001	0.003±0.001
	**Total**	15.36	21.134±0.167	0.936±0.007	1.058±0.002	0.059±0.002	0.037±0.001	0.038±0.001

No. of polymorphic alleles (N), No. of Different Alleles (Na), No. of Effective Alleles (Ne, = 1/(Sum pi∧2)), Shannon’s Information Index (I = −1* Sum (pi * Ln (pi))), Expected Heterozygosity (He = 1−Sum pi∧2) and Unbiased Expected Heterozygosity (UHe = (2N/(2N−1)) * He).

### Genetic diversity among the cultivars from different geographic regions

**Figure 5:**
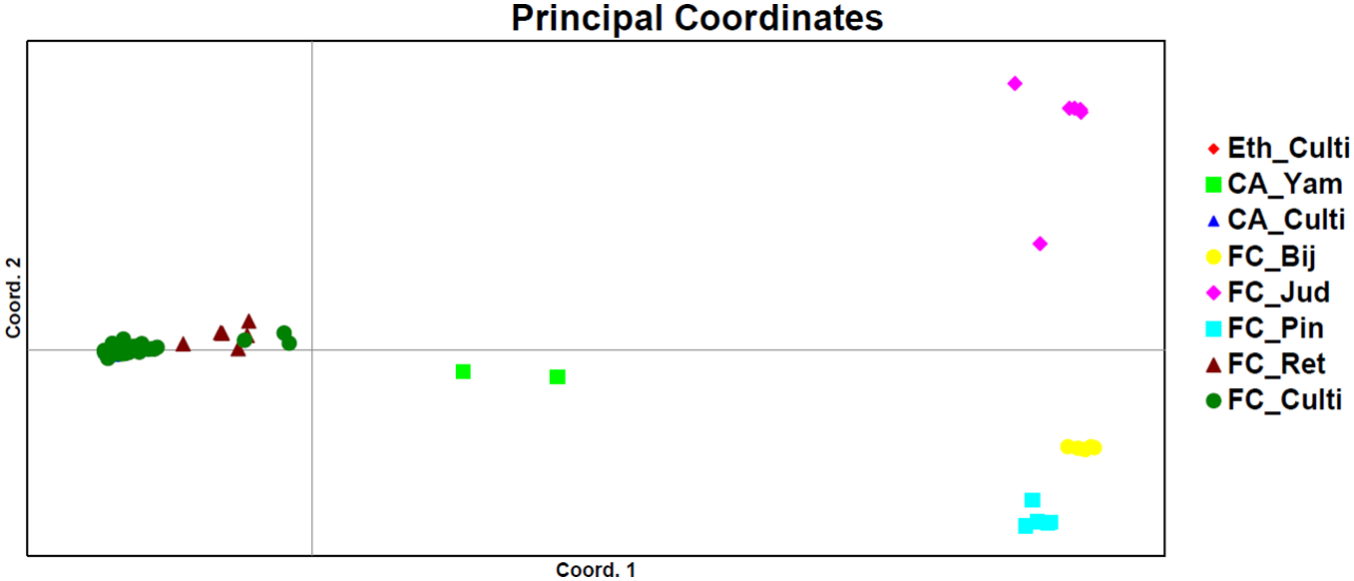
Principal coordinates analysis of wild and cultivated species of chickpea based on their geographical distribution (Eth_Culti: Cultivated chickpea from Ethiopia; CA_Yam: *Cicer yamashatae* from Central Asia; CA_culti: Cultivated chickpea from Central Asia; FC_Bij:, *C. bijugum*; from Fertile Crescent; FC_Jud: *C. judaicum* from Fertile Crescent; FC_Pin: *C. pinnatifidum* from Fertile Crescent; FC_Ret: *C. reticulatum* from Fertile Crescent and FC_Culti: Cultivated chickpea from Fertile Crescent).

To understand the diversity in chickpea cultivars from different regions, an analysis was also performed based on the geographical distribution of cultivated and wild species genotypes. Based on geographical origin, germplasm was divided in three clusters: the Fertile Crescent, Central Asia, and the Ethiopian highlands. Substantial geographic variation was observed, with the greatest diversity found in the Fertile Crescent and much lower diversity in the Ethiopian highlands and central Asia (Table 4). In parallel, PCoA was also performed (Figure 5). Outside of the Fertile Crescent, wild and cultivated material did not cluster together, which is consistent with a single domestication in the Fertile Crescent followed by dispersal to Central and South Asia and the East African highlands and subsequent divergence (Figure 5).

**Table 4:** Genetic variation across the three primary regions of diversity: Fertile Crescent, Central Asia, and the Ethiopian highlands using DArT markers.

	Polymorphic marker (%)	N	Na	Ne	I	He	UHe
**Ethiopia**	49.84%	5.895±0.007	1.467±0.011	1.206±0.004	0.232±0.004	0.145±0.003	0.175±0.003
**Central Asia**	54.11%	12.840±0.008	1.527±0.010	1.277±0.006	0.267±0.005	0.174±0.003	0.189±0.004
**Fertile Crescent**	98.62%	71.810±0.047	1.986±0.002	1.415±0.004	0.439±0.003	0.277±0.002	0.281±0.002

No. of polymorphic alleles (N), No. of Different Alleles (Na), No. of Effective Alleles (Ne, = 1/(Sum pi∧2)), Shannon’s Information Index (I = −1* Sum (pi * Ln (pi))), Expected Heterozygosity (He = 1−Sum pi∧2) and Unbiased Expected Heterozygosity (UHe = (2N/(2N−1)) * He).

## Discussion

Chickpea is believed to have been domesticated 10,000 years ago in southeastern Turkey and adjoining Syria [40]–[42]. The crop suffers from a narrow genetic base among the cultivated germplasm, which may be due to four population bottlenecks the crop has experienced [1]. This low genetic diversity makes the crop more susceptible to a range of diseases and pests [1], [17]. Recently, Varshney et al. [43] also confirmed the problem of narrow diversity in elite chickpea using whole genome re-sequencing of 90 chickpea lines. Wild relatives of chickpea could serve an important role in enhancing the genetic base of cultivated material. In an effort to understand the genetic diversity available in cultivated and wild gene pools, the present study was undertaken using SNP and DArT markers. Genetic diversity was analyzed for these loci across a panel of domesticated and wild germplasm in the ICRISAT collection [30].

Understanding the available genetic diversity in the germplasm collection is a pre-requisite to adopt effective conservation and management strategies to use these genetic resources in crop improvement. Understanding patterns of genetic diversity can complement efforts to match collections from differing climatic regions to planting zones differing in climate [24]. The present study focuses on exploration of the genetic diversity and population structure of this diverse set of chickpea that includes cultivated and wild species genotypes ranging from primary to tertiary gene pools [12]. Global research efforts have resulted in the development of a large number of markers (SSR, SNPs, DArT) and genotyping platforms that can be used to study genetic diversity and explore the diverse germplasm for the traits to use in chickpea improvement programs [44]. KASPar assay from KBiosciences (Hertfordshire, UK) (http://www.kbioscience.co.uk) provides flexibility in use and have been proven successful for molecular breeding applications involving only few markers for genotyping a large number of segregating lines [45]–[47]. In the case of chickpea, more than 2,000 KASPar assay [28] and high density DArT array with 15,360 DArT clones have been developed [29]. The present study used a subset of 651 SNPs along with DArT arrays for genotyping. SNP genotyping data was used for cultivated germplasm as alleles could not be called for most of the wild species genotypes. SNPs used in the present study were designed from cultivated chickpea, which may be the reason they could not be amplified in wild species and could contribute to the biomodel PIC values. SNPs, although powerful as a marker due to their declining costs and high number [28], can be biased by being developed from a small number of individuals. This bias can skew the pool towards older and more intermediate frequency SNPs [48], [49]. The benefit remains the large number of low cost markers. We minimized any effect of SNP bias by restricting its usage in the wild *Cicer* material where it lacks the information needed to separate patterns of relationships and complemented our analysis with the inclusion of independent DArT data that lacks such bias. In particular, focusing our analysis of the wild material on the DArT data should avoid the skew that SNP data can introduce.

In many crops that are deficient in genetic variation, wild relatives remain a critical resource. As is the case in other crops [4], [47], [50], higher levels of genetic variation were observed across all of the wild species. Significant genetic variation was observed in *C. reticulatum*, the immediate progenitor of cultivated chickpea, but genotypes of this species were less diverse than other *Cicer* species. Our results will allow the most genetically distinct of the existing accessions of these species to be used in breeding to maximize the diversity introgression into cultivated forms. However, as international germplasm collections contain only 18 unique *C. reticulatum* accessions [51], our results suggest that further collecting of *C. reticulatum*, particularly beyond the Mardin region of southeastern Anatolia where most existing collections were made, would be greatly beneficial. Relatively higher levels of genetic variation were present in the wild species of the secondary and tertiary gene pools, which span a far greater ecological range than *C. reticulatum,* which is restricted to oak savannas and disturbed pastures in southeastern Anatolia. However, the levels of genetic variation were still not all that high, consistent with the high probabilities on the assignment tests and the primarily selfing reproductive system of most *Cicer* species. Traits of wild species that are beneficial in a Mediterranean climate, such as vernalization, can hinder efforts to breed chickpea for cultivation in subtropical climates. Therefore, wild species from different regions, such as the African highlands or Central Asia could provide climatically adaptive traits for chickpea production in non-Mediterranean climates. For instance, species from outside the Fertile Crescent, such as *C. cuneatum* from Ethiopia and *C. microphyllum* from Central Asia (Pakistan and Afghanistan) could be exploited as sources of adaptive variation for those regions. Furthermore, wild species from more arid environments, such as *C. judaicum* and *C. pinnatifidum,* could be useful in expanding the resistance of cultivated chickpea to important biotic stresses like *Ascochyta*, *Helicoverpa, Fusarium* and *Botrytis* Gray Mold [20].

Based on seed type, chickpea has been subdivided in to three groups: desi, kabuli and pea-shaped. Significant differentiation among desi and kabuli seed type cultivars was observed, although far less than exists between wild species. The distinction could be due to a relatively recent evolution of kabuli seed type from a desi seed type ancestor that closely resembled the wild species, as previously speculated [16], but could just as easily represent artificial population structure generated by breeders [52]. Regardless, the division between the phenotypic classes of seed type appears to be weak and likely of recent origin. The dearth of desi seed type genotypes from the Fertile Crescent could suggest that kabuli seed types were favoured in this region, potentially as a means to prevent introgression from *C. reticulatum* and *C. echinospermum,* which have seed and flower colours similar to desi seed types.

Germplasm collections contain relatively low numbers of wild relatives of crops [6]. Although often several individual lines of a wild species are available, rarely has collecting been aimed at understanding patterns of variation in populations of wild relatives [53], [54]. Our results indicate that collecting diverse population samples of several *Cicer* species spanning ecologically meaningful gradients in abiotic or biotic factors such as moisture, soil fertility or pathogen distribution would be extremely useful. Analysis of variation across these gradients in wild relatives could show how natural selection has adapted populations of wild relatives to these localized conditions, giving us natural targets for breeding.

## Supporting Information

Figure S1 a. Principal coordinates analysis of wild species of chickpea based on primary, secondary and tertiary gene pool. b. Population structure analysis across wild chickpea accessions to understand the distribution of primary, secondary and tertiary gene pool species.
(TIF)

Figure S2 a. Population structure analysis across cultivated chickpea accessions based on seed type. b. Analysis of molecular variance within and among cultivated population based on seed type.
(TIF)

Table S1 **Details about the Cicer accessions used in the study.**
(XLS)

Table S2 **Summary of the genotyping data generated using 651 CKAM markers on 94 Cicer accessions.**
(XLS)

Table S3 **Summary of the genotyping data generated using DArT markers.**
(XLS)
