# Supplementary figures and images for: Exploring Germplasm Diversity to Understand the Domestication Process in Cicer spp. Using SNP and DArT Markers
Source: PLoS One. 2014 Jul 10;9(7):e102016. doi: 10.1371/journal.pone.0102016 (PMC4092095; doi:10.1371/journal.pone.0102016)

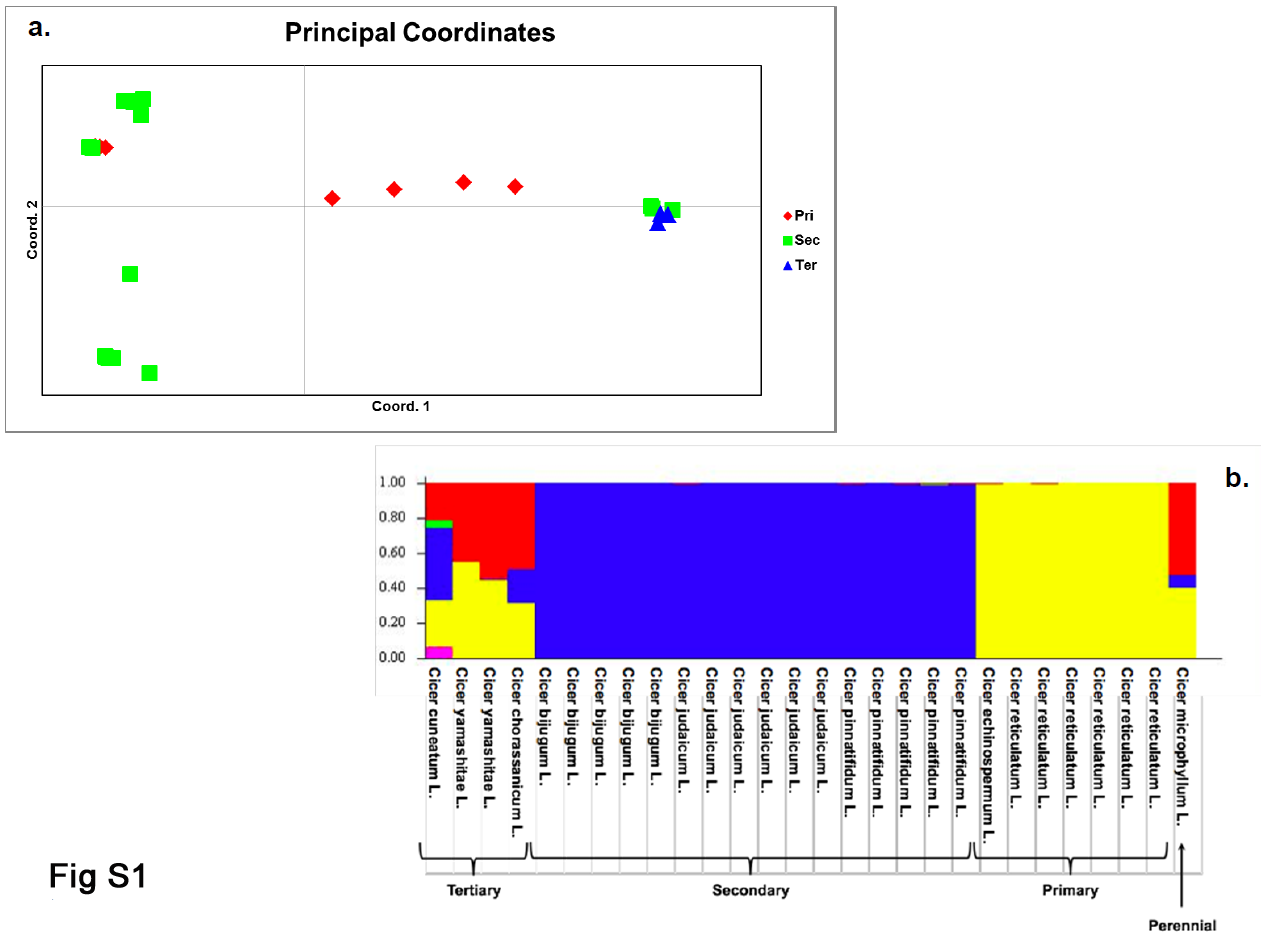

Supplement: Figure S1 — a. Principal coordinates analysis of wild species of chickpea based on primary, secondary and tertiary gene pool. b. Population structure analysis across wild chickpea accessions to understand the distribution of primary, secondary and tertiary gene pool species. (TIF) [file pone.0102016.s001.tif]

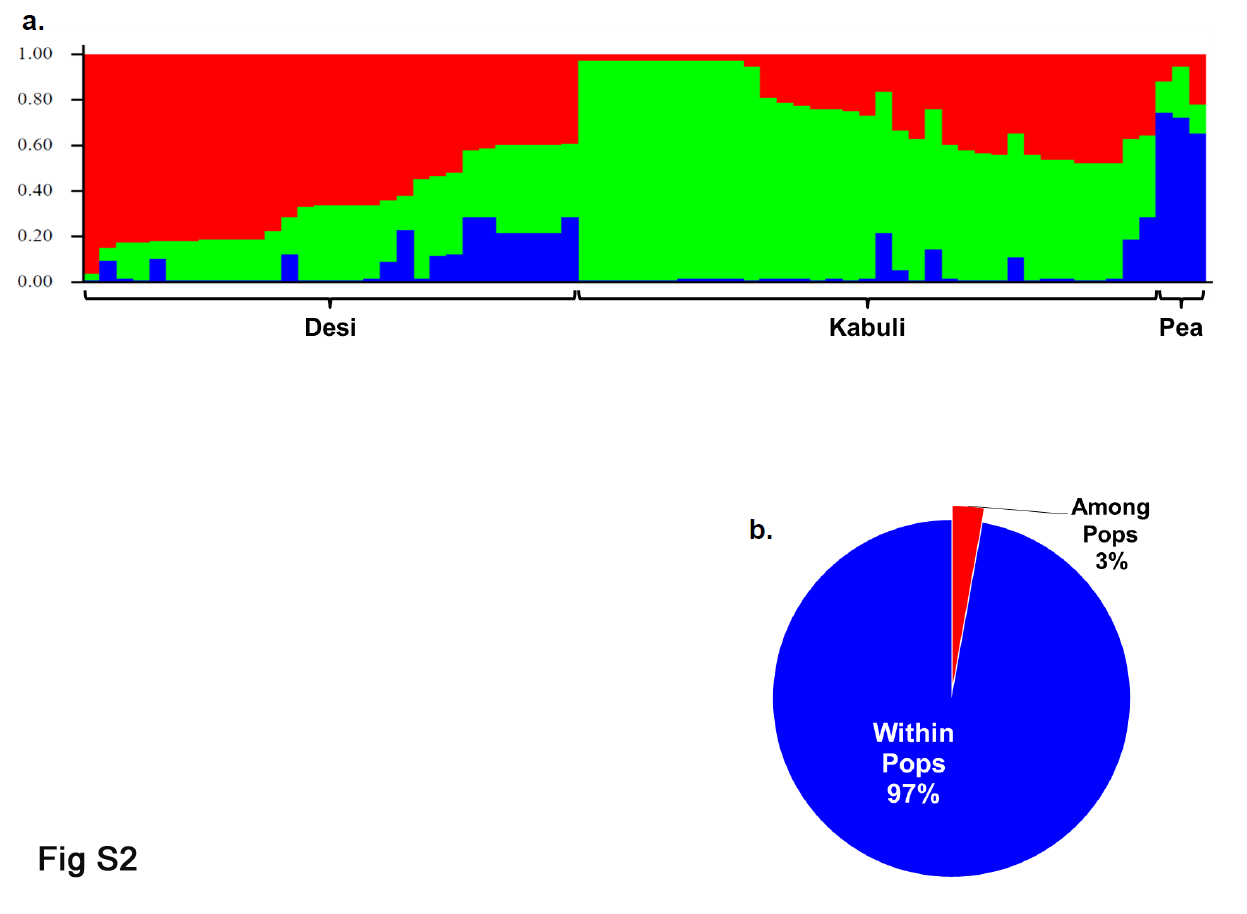

Supplement: Figure S2 — a. Population structure analysis across cultivated chickpea accessions based on seed type. b. Analysis of molecular variance within and among cultivated population based on seed type. (TIF) [file pone.0102016.s002.tif]
